# Reduced‐Port Laparoscopic Colectomy for Colorectal Cancer: The Comparison That Matters Now Is With Reduced‐Access Robotic Surgery

**DOI:** 10.1002/ags3.70273

**Published:** 2026-08-31

**Authors:** Mehmet Mahir Özmen, Cem Kaan Parsak, Tahsin Çolak

**Affiliations:** ^1^ Department of Surgery Liv Hospital Ankara Turkey; ^2^ MOC Obesity and Cancer Clinic Ankara Turkey; ^3^ Department of General Surgery, Division of Surgical Oncology Çukurova University Faculty of Medicine Adana Turkey; ^4^ Department of General Surgery, Division of Colorectal Surgery Mersin University Faculty of Medicine Mersin Turkey

We read the systematic review and meta‐analysis by Shaukat et al. with interest [1]. It is, to our knowledge, the first meta‐analysis to compare reduced‐port laparoscopic colectomy (RPLC) with conventional multi‐port laparoscopic colectomy (MPLC) specifically for colorectal cancer, and the methodology is sound: a PRISMA‐adherent, prospectively registered protocol, and a meta‐regression that correctly identifies tumor stage as the main driver of the operative‐time benefit and of the between‐study heterogeneity.

Our main comment concerns what the comparison leaves out. Excluding robotic‐assisted procedures is defensible for internal comparability, but it sidesteps the question that is becoming more relevant every year. Single‐port and reduced‐port robotic platforms are already in clinical use for colorectal resection, with early series reporting acceptable oncologic and short‐term outcomes [2]. A scoping review of that literature published earlier this year found reporting just as fragmented as the pattern the authors describe for laparoscopic RPLC: only 9 of 19 studies used a standardized complication‐grading system, and follow‐up interval was documented in barely a quarter of them [3]. For a department now deciding where to invest its training effort and capital, RPLC versus reduced‐access robotic surgery is the more pressing comparison—conventional five‐port laparoscopy is no longer the only alternative on the table.

A smaller point: the higher rate of Clavien–Dindo Grade 1 complications in the RPLC arm, despite fewer incisions, may reflect a genuine learning‐curve effect rather than ascertainment bias alone, given that “RPLC” here pools two‐port, three‐port, and solo‐surgeon configurations under one label.

Both issues point to the same fix: neither laparoscopic nor robotic RPLC will be comparable across studies, or against each other, until they are reported against a common minimum dataset (Table 1).

**TABLE 1:** Minimum reporting elements proposed for future RPLC studies.

Domain	Reporting element
Technique	Port number, size, and location; solo‐surgeon versus assisted setup; laparoscopic versus robotic platform
Surgeon factors	Case volume and learning‐curve stage at time of series
Case mix	Tumor stage, BMI, T4 disease, prior abdominal surgery
Safety	Complication grading system and surveillance/reporting protocol
Oncologic outcome	Recurrence, disease‐free and overall survival with follow‐up duration

Current guidelines endorse minimally invasive resection for colorectal cancer without specifying a preferred port configuration [4], leaving this choice to individual surgeon and institutional experience. Registries run jointly by minimally invasive and colorectal surgical societies, built around this dataset, would let future analyses adjust for the confounders the authors already flag—and would let RPLC be tested against reduced‐access robotic surgery on equal terms, not only against five‐port laparoscopy.

## Author Contributions

Letter to the editor is prepared by M.M.Ö. and evaluated and corrected by M.M.Ö., C.K.P., and T.Ç.

## Funding

The authors have nothing to report.

## Conflicts of Interest

The authors declare no conflicts of interest.

## Data Availability

The data that support the findings of this study are available from the corresponding author upon reasonable request.
